# Characterisation of the protein corona using tunable resistive pulse sensing: determining the change and distribution of a particle’s surface charge

**DOI:** 10.1007/s00216-016-9678-6

**Published:** 2016-06-10

**Authors:** Emma L. C. J. Blundell, Matthew J. Healey, Elizabeth Holton, Muttuswamy Sivakumaran, Sarabjit Manstana, Mark Platt

**Affiliations:** 1 Department of Chemistry, Loughborough University, Loughborough, LE11 3TU UK; 2 Peterborough City Hospital, Edith Cavell Campus, Bretton Gate, Peterborough, PE3 9GZ UK; 3 Human Genomics Lab, Centre for Global Health and Human Development, School of Sport, Exercise and Health Sciences, Loughborough University, Loughborough, UK

**Keywords:** Biosensor, TRPS, Zeta potential, Protein corona, Tunable pores

## Abstract

The zeta potential of the protein corona around carboxyl particles has been measured using tunable resistive pulse sensing (TRPS). A simple and rapid assay for characterising zeta potentials within buffer, serum and plasma is presented monitoring the change, magnitude and distribution of proteins on the particle surface. First, we measure the change in zeta potential of carboxyl-functionalised nanoparticles in solutions that contain biologically relevant concentrations of individual proteins, typically constituted in plasma and serum, and observe a significant difference in distributions and zeta values between room temperature and 37 °C assays. The effect is protein dependent, and the largest difference between the two temperatures is recorded for the γ-globulin protein where the mean zeta potential changes from −16.7 to −9.0 mV for 25 and 37 °C, respectively. This method is further applied to monitor particles placed into serum and/or plasma. A temperature-dependent change is again observed with serum showing a 4.9 mV difference in zeta potential between samples incubated at 25 and 37 °C; this shift was larger than that observed for samples in plasma (0.4 mV). Finally, we monitor the kinetics of the corona reorientation for particles initially placed into serum and then adding 5 % (*V*/*V*) plasma. The technology presented offers an interesting insight into protein corona structure and kinetics of formation measured in biologically relevant solutions, i.e. high protein, high salt levels, and its particle-by-particle analysis gives a measure of the distribution of particle zeta potential that may offer a better understanding of the behaviour of nanoparticles in solution.

**Electronic supplementary material:**

The online version of this article (doi:10.1007/s00216-016-9678-6) contains supplementary material, which is available to authorized users.

**Graphical Abstract:**
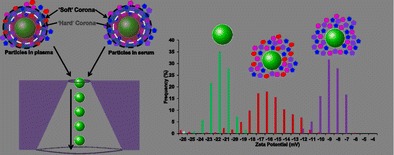
The relative velocity of a nanoparticle as it traverses a nanopore can be used to determine its zeta potential. Monitoring the changes in translocation speeds can therefore be used to follow changes to the surface chemistry/composition of 210 nm particles that were placed into protein rich solutions, serum and plasma. The particle-by-particle measurements allow the zeta potential and distribution of the particles to be characterised, illustrating the effects of protein concentration and temperature on the protein corona. When placed into a solution containing a mixture of proteins, the affinity of the protein to the particle’s surface determines the corona structure, and is not dependent on the protein concentration

## Introduction

In recent years, synthesis methods for nanoparticles have evolved to the extent that particle size, shape and composition can be easily modified [1–4] and this had led in turn to great advances in the field of diagnostics [5, 6], drug delivery [7–9] and technology platforms [10, 11]. With the desire to understand and improve nanomaterials comes a need for characterisation platforms to offer rapid analysis of size, charge and shape. Ensemble techniques that take measurements on several particles simultaneously and provide an average measurement can underestimate subpopulations within multimodal samples [12, 13], and a raft of technologies have appeared to help tackle this [14, 15]. Such technologies now offer an ability to quantify the population of particles with single particle resolution building an understanding that not all particles are created equal and there exists distributions such as particle size or ligand density.

**Fig. 1:**
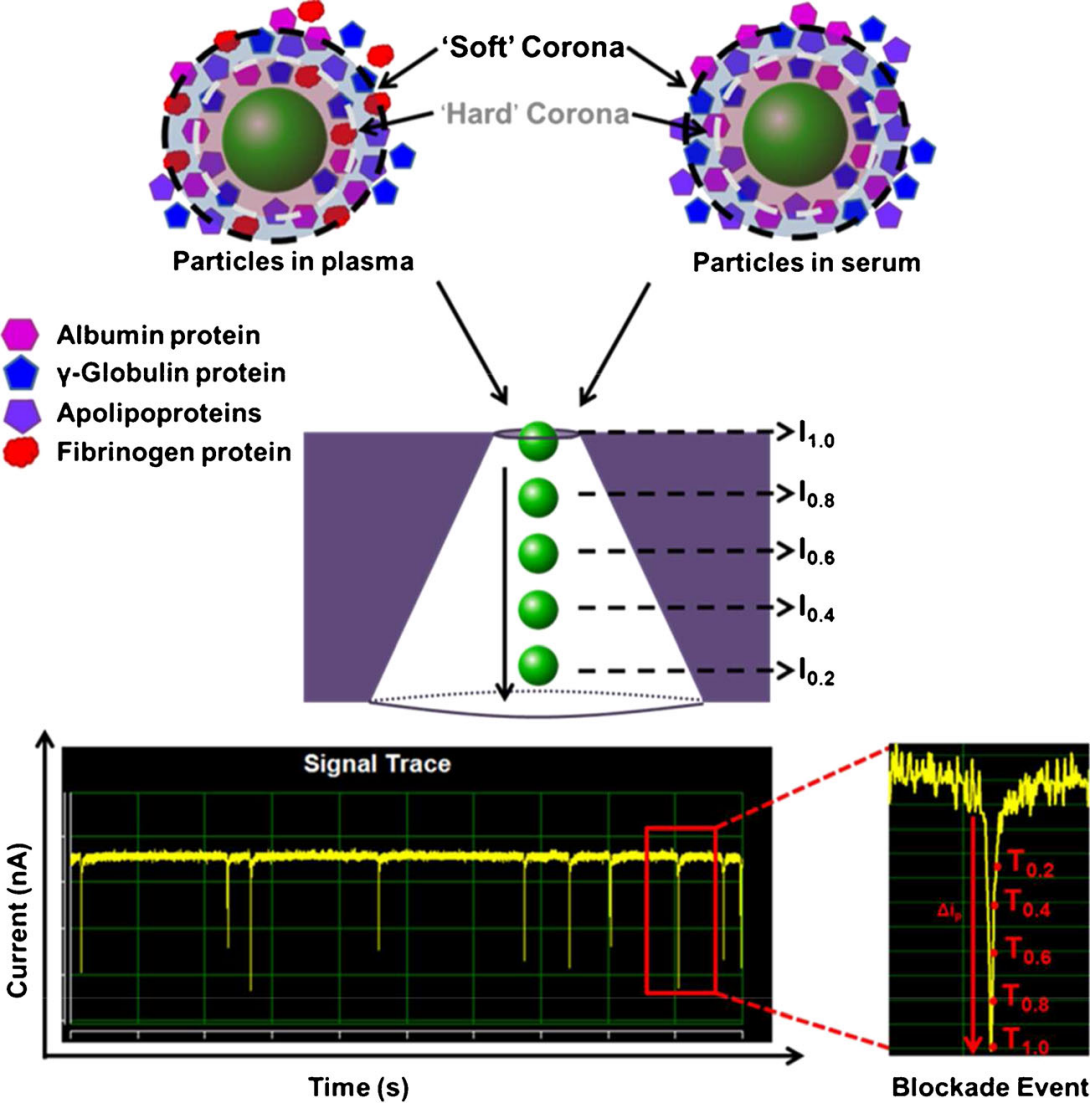
Particles in the presence of human plasma and serum showing the formation of both a ‘hard’ and ‘soft’ protein corona. I_1.0_, I_0.8_, I_0.6_, I_0.4_, I_0.2_ represent the position of the particle as it translocates the pore (where I_1.0_ is the narrow pore entrance) and are relative to T_1.0_, T_0.8_, T_0.6_, T_0.4_, T_0.2_, which represent the time taken (ms) for the particle to reach that position. T_1.0_, is equivalent to dR_max_ when the blockade event is at 100 % magnitude; T_0.8_, T_0.6_, T_0.4_, T_0.2_, correspond to when the blockade is 80, 60, 40, and 20 % of its dR_max_ and indicates the particle traversing the pore

One such technology is based on the Coulter Counter principle, referred to as resistive pulse sensing (RPS) [16–18]. The technique allows the characterisation of proteins, inorganic ions, colloids and nanoparticles within their natural environment. Two categories of resistive pulse sensors exist that utilise either biological [19, 20] or inorganic nanopores [21–23]. Here, we describe a recent adaptation to inorganic pores that uses a tunable elastomeric pore termed tunable resistive pulse sensing (TRPS) [14, 24–36]; the pore can be stretched in real time to suit the sample. The brief setup and theory for TRPS technologies is as follows: A stable ionic current is established by two electrodes, separated by a pore; as particles/analytes translocate the pore, they temporarily occlude ions, leading to a transient decrease in current known as a ‘blockade event’, examples of which can be seen in Fig. 1. In the TRPS arrangement used here, the pore is mounted laterally so that particles typically move from the upper fluid cell into the lower fluid cell, aided by an inherent pressure head due to 40 μl of liquid in the upper fluid cell of approximately 50 Pa [35]. By monitoring changes in blockade width, blockade magnitude (*Δi*_*p*_) and blockade frequency (events/min), it is possible to elucidate the zeta potential, size and concentration of colloidal dispersions in situ [14, 37, 38]. By controlling the aspect ratio of the pore, resistive pulse sensors have been used to measure analytes that range from single molecules, DNA, proteins, cellular vesicles to cell bacteria and viruses; detailed reviews on the types of analytes and applications can be found elsewhere [24, 36, 39, 40]. TRPS is becoming an increasingly common variation of RPS for the characterisation of biological and inorganic nanomaterials [24, 36] and since its conception has been tested against alternative technologies such as DLS/PALS [14, 15, 41–44], TEM [33], and ultracentrifugation [44] for the characterisation of nanomaterials [15, 45].

The *how* and *where* of measuring the properties of particles are important to consider as changing pH, ionic strength or temperature, or purifying particles can give a misrepresentation of their behaviour in their natural environment. In the case of nanomaterials that are intended to be used in vivo, it is not properties within synthesis processes that determine their biological activity, but how they interact with proteins upon entering the body. Upon the addition of nanoparticles to biological fluids, there is an almost immediate fouling of their surfaces with proteins, peptides and other cellular apparatus forming a layer known as the protein corona [46–48]. The composition of the corona has been shown to determine the eventual properties of the particles [49–51] and has been reported as critically affecting pathophysiological effects of nanoparticles [52]. The structure of the protein corona can be dynamic and complex and is different for particles of the same composition but with different surface chemistries and size in the same solution [46, 53]. Detailed studies of the corona have been performed using an array of technologies including mass spectroscopy [54, 55]. Various techniques have been used to look at a range of specificities of protein coronas, for example, protein corona thickness has been investigated using ensemble techniques such as dynamic light scattering (DLS) and differential centrifugal sedimentation (DCS) [49, 56]. Protein corona conformation has been studied using circular dichroism (CD) and fluorescence quenching [57, 58]; the affinity has also been a popular characterisation property of protein coronas and has previously been measured using size exclusion chromatography (SEC), surface plasmon resonance (SPR) and isothermal calorimetry (ITC) [49, 56, 59]. A frequent and easy value used to characterise the corona is zeta potential [46, 48, 50, 54, 60]. The zeta potential represents the value of the electrostatic potential at the plane of shear, and typically for nanoparticle systems, zeta potential values of ±30 mV are representative of stabilised particles [61].

When nanoparticles are introduced to biological fluids, the protein corona is formed in a series of layers, otherwise known as the ‘hard’ and ‘soft’ corona. Proteins forming the hard corona are those with a higher affinity that interact directly with the nanoparticle surfaces, whereas proteins forming the soft corona are those engaging in weaker protein–protein interactions with the hard corona [56, 62]. It has previously been found that a vast range of particles bind successfully to apolipoproteins in physiological fluids [55]. Formation of a protein corona alters the size, aggregation properties and surface properties of nanoparticles [63], thus creating a new biological distinctiveness for further application. There are 5 main components that define the composition of a protein corona: thickness and density, identity and quantity, orientation, conformation and affinities [63].

Protein adsorption kinetics play a prominent role in this study and are key to understanding the binding mechanisms that will occur in a natural environment. Although this process is time-dependent, the kinetics rely on *k*_on_ and *k*_off_ parameters, indicating the rate constants for adsorption and desorption of proteins. *k*_on_ is largely dependent on how often the protein contacts the nanoparticle surface, as well as the probability of successful binding between the two materials [64]. The strength of the protein–nanoparticle interaction defines *k*_off_ [64], and a strong, high-energy interaction will exert a low *k*_off_ value. Understanding the kinetics of formation and protein corona composition is important to understand processes nanoparticles may undertake when introduced into the body and into physiological conditions.

Here, we present a protocol for the rapid analysis of the corona zeta potential and demonstrate its versatility by making the measurement in solutions that mimic the natural environment, i.e. high ionic strength and high protein composition. By making comparable measurements of carboxyl polystyrene nanoparticles in a range of incubation temperatures and with different proteins, a clear difference in magnitude and variation of zeta potential within the particle population was observed; the three proteins chosen to demonstrate this are the most predominant (in terms of quantity) proteins in normal human plasma and we perform the experiment concentrations that would reflect normal plasma. The ability to have individual particle resolution provides an opportunity to see the full variation of zeta potential in a single sample. The findings highlight the need to monitor the protein corona and its formation at biologically relevant temperatures and suggest that the kinetics of protein adsorption and spread in zeta potential values varies for each of the proteins and biological mediums studied. Finally, we show the scope of the technology by monitoring the change in the hard and soft corona elements interacting with the particles through incubation in serum, followed by the addition of a small amount (5 % (*V*/*V*)) of plasma. It is known that protein components of a higher concentration or affinity to the particle can remove and restructure the soft corona that is formed in biological fluid [55], and we monitor the rate of this change and the kinetic effects that eventually settle on a new zeta potential value.

## Materials and methods

### Chemicals and reagents

The initial buffer used for particle analysis was phosphate buffered saline (1× PBS tablet (0.01 M phosphate buffer, 0.0027 M potassium chloride, 0.137 M sodium chloride) in 200 mL deionised water (18.2 MΩ cm)). PBS tablets (P4417) were purchased from Sigma-Aldrich, UK.

#### Carboxyl polystyrene standards

Carboxylated polystyrene particles, denoted as CPC200, with a mean nominal diameter of 210 nm and stock concentration of 1 × 10^12^ particles/mL, were purchased from Bangs Laboratories, USA and used as a calibrant for zeta potential analysis, as well as the sample particles. CPC200s were vortexed for 30 s followed by a 2 min sonication to ensure monodispersity prior to any TRPS analysis or sample incubation.

#### Isolated proteins

All isolated proteins studied were purchased from Sigma-Aldrich, UK, without modification or purification unless stated otherwise: fibrinogen from human plasma (F3879), albumin from human serum (A9511) and γ-globulin from human blood (G4386).

#### Human plasma and serum samples

Blood samples were collected and prepared at Peterborough City Hospital Pathology Laboratory, UK. Plasma collection was completed using blood from a healthy volunteer donor that was collected in citrate medium (Sarstedt, UK.) and centrifuged at 3000 rpm for 8 min. Serum was gathered using blood from a healthy volunteer donor that was collected into a Sarstedt monovette/collection tube, and was centrifuged at 3000 rpm for 6 min. The supernatants from each sample were transferred into separate sample vials and stored at room temperature prior to use.

#### Isolated protein studies

Using PBS buffer, isolated albumin, fibrinogen and γ-globulin samples were prepared to give the following concentrations: 43, 3.2 and 20 g/L, respectively, as to mimic protein concentrations found in human blood. The concentrations of proteins were measured from human plasma and serum samples. The samples used in this study were analysed by an Instrument Laboratory ACL TOP CTS500 coagulation analyser (Werfen, Spain) to obtain the fibrinogen concentration. Albumin and immunoglobulin levels were taken from test serum samples that were analysed by a Roche Cobas Biochemistry Analyser (Roche Diagnostics, Switzerland). CPC200s were added resulting in a final concentration of 1 × 10^10^ particles/mL. Each sample was vortexed for 30 s and sonicated for 1 min before incubation. Samples were then incubated at 25 and 37 °C in a mini dry bath (Benchmark Scientific, USA) for 10 min prior to TRPS analysis.

#### Serum and plasma studies

Human plasma and serum were prepared immediately before the experiments to minimise ex vivo artefactual changes. The prepared plasma and serum were separately diluted 10-fold with PBS before CPC200s were added to both samples resulting in a final particle concentration of 1 × 10^10^ particles/mL, herein these solutions are referred to as serum and plasma. Samples were vortexed for 30 s and sonicated for 1 min, followed by incubation in a mini dry bath (Benchmark Scientific, USA) at 25 and 37 °C for 10 min before being removed for TRPS analysis. It should be noted that it is possible for some proteins in human plasma and serum to interact and adsorb onto the pore walls; therefore, a control measurement of CPC200s in PBS (of known zeta potential, -20 mV) was completed before and after each protein/plasma/serum sample to establish if any changes had occurred to the pore itself.

#### Plasma spiking assay

Human serum was 10× diluted in PBS before CPC200s were added to a final concentration of 1 × 10^10^ particles/mL. Samples were vortexed for 30 s and sonicated for 1 min before being incubated for 10 min at 25 and 37 °C in a mini dry bath (Benchmark Scientific, USA). At 10 min, 5 % (*V/V*) human plasma was added to the serum samples and the samples were vortexed for 30 s. TRPS measurements were completed once the plasma had incubated with the serum sample for 5, 10, 15, 20, 30 and 60 min.

#### Tunable resistive pulse sensing

All measurements were completed using the qNano (Izon Science Ltd, NZ). The system utilises tunable nanopores with propriety data capturing software (Izon Control Suite v3.1.2.53). In all experiments, the lower fluid cell contained 80 μL of PBS buffer, ensuring no bubbles were present. When a sample measurement was being taken, the upper fluid cell contained 40 μL of the sample (suspended in PBS buffer). After each measurement was taken, the nanopore was washed several times by removing and replacing 40 μL of buffer, each time applying varied pressures until no particles were observed. This was performed several times to remove any residual particles in the system and thus ensure no cross-contamination between samples. The nanopores used throughout all experiments were capable of detecting particles within the size range of 100–300 nm (as stated by the manufacturer, Izon Science Ltd) and denoted as an NP200. To account for the variation in the manufacturing of the nanopores, appropriate stretch (44–46 mm), voltage and pressure were applied in all experiments; the conditions were matched as to the blockade magnitudes of CPC200s in PBS being of a similar size throughout all experiments. All samples were vortexed for 30 s and sonicated for 2 min prior to analysis.

#### Zeta potential measurements using TRPS

When carrying out zeta potential measurements, the nanopore stretch was kept the same for a particular dataset and nanopore between calibration and sample measurements. To calibrate a nanopore for zeta potential analysis, the calibration particles, of known size and zeta potential, were measured in PBS at 3 applied voltages; the particles measured at the highest voltage were measured at 2 external pressures (in addition to the inherent 47 Pa pressure head on the system). When running the samples, the blockade magnitudes were ensured to be at least 100× larger than the respective background noise of ca. 10 pA. In accordance with the calibration runs, the samples were run at the highest calibration voltage. Calibration measurements were completed when a new nanopore (NP200) was introduced to ensure conditions were matched so the blockade magnitudes of CPC200s in PBS were of a similar size to other NP200s used for this study. A CPC200 sample in PBS was run after each protein/plasma/serum sample to ensure the zeta potential of the pore remained unchanged and as such did not affect the measured zeta potential of further samples.

## Results and discussion

Zeta potential values were determined from the particle velocities as they traversed the nanopore; a full description of the protocol and theory can be found elsewhere [21, 38]. Briefly, the duration of particle translocation is measured as a function of applied voltage, taking an average electric field and average particle velocities over the entire sensing zone that is a regular conical pore. Each particle’s electrophoretic mobility is derived from *1/T*, where *T* is the blockade duration and voltage, multiplied by the square of the sensing zone length, *L*, as part of a calibration constant. Figure 1 shows the conical sensing zone and an example of the blockade duration times, *T*, as a result of a blockade event at various positions, *I*, in the nanopore. *T*_*1.0*_ for example is equivalent to when the blockade is 100 % in magnitude and is indicative of *I*_*1.0*_, the position to which the particle is approaching the pore entrance. *T*_*0.6*_ relates to position *I*_*0.6*_ where the blockade is 60 % in magnitude and the particle has traversed 40 % of the pore. It is important to note each blockade depicted in the signal trace is indicative of a single particle as it passes through the pore, highlighting the advantages of using particle-by-particle technologies such as TRPS.

Average velocities determined across multiple reference points within the nanopore vastly reduce any errors in this zeta potential calculation process [38]. The calibration of the pore itself is based on a linear relationship between *1/T* and voltage, *V*, at each blockade reference point. Equation 1 shows the direct relationship between particle velocities and their zeta potentials, ${\left({v}_x\right)}_{el\;Cal}$ and ${\left({v}_x^i\right)}_{el\; Sample}$ are the particle velocities of calibration and sample particles, respectively, and ${\xi}_{net\;Cal}$ and ${\xi}_{x\;net\; Sample}^i$ represent their zeta potential values [38].

1 $$ \frac{{\left({v}_x^i\right)}_{el\kern0.5em  Sample}}{{\left({v}_x\right)}_{el\kern0.5em Cal}}=\frac{{\xi_x^i}_{\kern0.5em net\kern0.5em  Sample}}{\xi_{net\kern0.5em Cal}} $$

Equation 2 shows the zeta potentials measured at each of the blockade reference points can then be used to determine the zeta potential of each individual particle, *i*, as it passes through the pore, ${\xi}_{Sample}^i$.

2 $$ {\upxi}_{\mathrm{Sample}}^{\mathrm{i}}=\frac{\sum_{\mathrm{x}}{\upxi}_{\mathrm{x}\kern0.5em \mathrm{Sample}}^{\mathrm{i}}}{\sum_{\mathrm{x}}}=\frac{\sum_{\mathrm{x}}\left({\mathrm{v}}_{\mathrm{x}\kern0.5em \mathrm{Sample}}^{\mathrm{i}}-{\mathrm{v}}_{\mathrm{x}\kern0.5em \mathrm{C}\mathrm{a}\mathrm{l}}^{\mathrm{P}}\times P\right)/\left({\mathrm{v}}_{\mathrm{x}\kern0.5em \mathrm{C}\mathrm{a}\mathrm{l}}^{\mathrm{V}}\times V\right)}{\sum_{\mathrm{x}}}\times {\upxi}_{\mathrm{net}\kern0.5em \mathrm{C}\mathrm{a}\mathrm{l}}+{\upxi}_{\mathrm{m}} $$

Where ${{\mathrm{v}}_{\mathrm{x}}^{\mathrm{V}}}_{\mathrm{Cal}}$,${{\mathrm{v}}_{\mathrm{x}}^{\mathrm{P}}}_{\mathrm{Cal}}$, *P* and *V* are electrokinetic velocity per unit voltage, convective velocity per unit pressure, applied pressure and voltage, respectively. *I*_*x*_ is the position of the particle in the nanopore after time *t = T*_*x*_ *, v*_*x Sample*_^*i*^ is the sum of the particle velocities at relative positions, *l*_*x*_ [38].

**Fig. 2:**
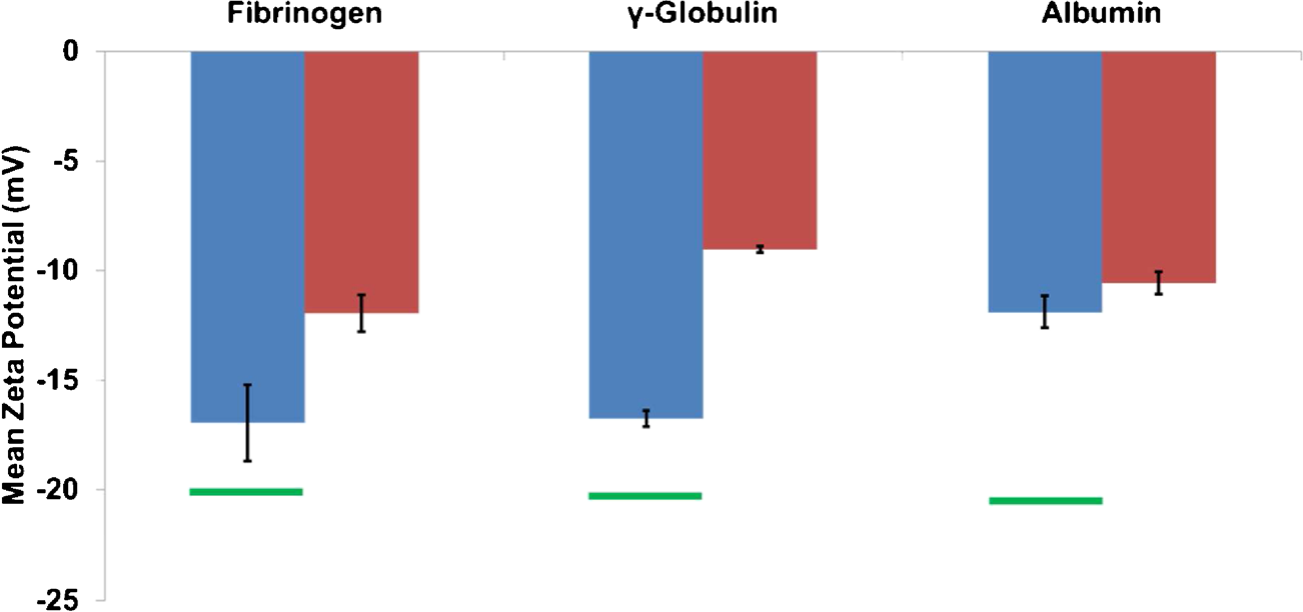
Mean zeta potential (mV) versus the protein the particles were incubated with. The *blue bars* show results for a 10-min particle incubation at 25 °C and the *red bars* show the mean zeta potential values for particles incubated with the proteins for 10 min at 37 °C. The *green lines* represent the measured mean zeta potential for calibration particles of known zeta potential (−20 mV) in PBS that were run after each protein sample to show the protein samples were not having a direct effect on the pore walls themselves that may influence the recorded zeta potentials of future samples run on the same pore. *Error bars* are representative of the st.dev where *n* = 3

The proteins used in this study were chosen based on their relative abundances in both blood plasma and serum samples. Albumin and γ-globulin are present in both plasma and serum samples at approximately 4 and 2 %, whereas fibrinogen (as well as other clotting factors) is only found in plasma at approximately 0.4 %. Zeta potential values measured for particles incubated with each of the isolated proteins are shown in Fig. 2 (for reference the starting zeta potential of a CPC200 in PBS is −20 mV).

**Fig. 3:**
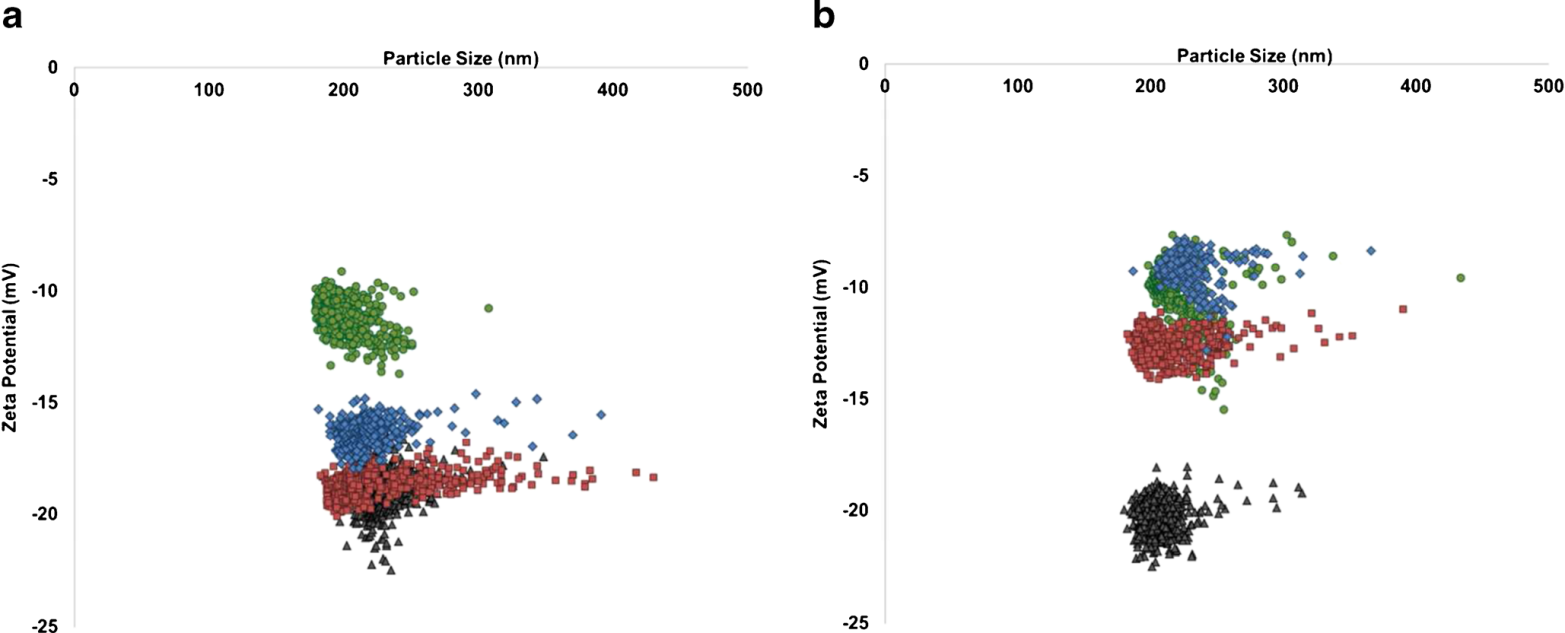
Zeta potential (mV) versus particle size (nm). The *red*, *blue* and *green* datasets are zeta potential distributions for CPC200s incubated for 10 min with fibrinogen, γ-globulin and albumin, respectively at (**a**) 25 °C and (**b**) 37 °C. The *black data points* represent CPC200s in PBS for both figure parts **a** and **b**

When the particles were incubated with each of the proteins separately at 25 °C, both fibrinogen and γ-globulin showed a relatively small change in mean zeta potential from particles in PBS buffer, differences of 3.2 and 3.6 mV, respectively. The size and zeta potential distributions of CPC200 carboxyl particles in PBS are shown in Fig. 3. Albumin was seen to have a much larger effect on the particle zeta potentials at 25 °C as the zeta values were reduced by 9.2 mV from the PBS control. Albumin was at the highest concentration at 40 g/L in comparison to the fibrinogen and γ-globulin samples only having protein concentrations of 4 and 20 g/L respectively. The protein concentrations were chosen to replicate the typical composition usually found in the human body, although it should be noted that the concentration of proteins to that of the particles in each experiment was always in a large excess as to coat each surface of every particle. The proteins were also investigated at a constant concentration (5 g/L) at a 25 °C incubation temperature for 10 min to determine whether protein concentration had an effect on the protein corona on the particles, results of which are shown in the Electronic Supplementary Material (ESM), Fig. S1. From this, it was found that the relative change in zeta potential (from a control of the particles just in PBS) was smallest for fibrinogen and γ-globulin with values of 4.3 and 4.9 mV, respectively. The largest change in zeta potential was again observed for the albumin protein with a difference of 8.9 mV. These comparable changes show the results are protein specific and not related to the concentration at these levels. It was therefore expected that the proteins would adsorb onto the particle surface, forming the protein corona. Any such protein corona would change the surface charge density on the particles and be measured by a change in particle velocity, which in turn is plotted as the zeta potential. At 25 °C, the small zeta potential changes for fibrinogen and γ-globulin samples are more than likely because of the protein isoelectric points and their behaviour at physiological pH. Albumin has an isoelectric point of 4.7 whereas fibrinogen and γ-globulin have isolectric points of 5.8 and 6.6, respectively [65]. Previous reports have found that as the adsorption pH moves away from the protein isolectric points, the adsorbed molecules will occupy a larger area of the surface. This is due to internal electrostatic repulsions and thus a lower structural stability [66]. Our samples were all suspended in PBS buffer at pH 7.4, and therefore, the albumin is expected to occupy the largest area of the nanoparticle surface as the adsorption is occurring at a pH furthest from its isoelectric point. This may be the reason the albumin shows the largest change in zeta potential after a 25 °C incubation in comparison to the smaller changes observed for fibrinogen and γ-globulin (isoelectric points closer to 7.4).

Particles were also incubated with each of the proteins at a higher temperature of 37 °C; it was hypothesised that as the proteins are present in such a large excess that the incubation time of 10 min would be enough to coat the particles with a monolayer of protein, and that the temperature would have little effect on the result. In contrast, at 37 °C, there were significant differences from values at 25 °C and each protein produced varying shifts in zeta potential values. At the elevated temperature, γ-globulin was seen to have the largest reduction in zeta potential from a value of −20.3 mV (particles in PBS) to −9.0 mV. This is of particular interest as these results indicate each protein interacts with the particle surface uniquely, having direct implications on the particle zeta potential. γ-Globulin also showed the largest change in zeta potential as a function of incubation temperature between 25 and 37 °C (5.0 mV), whereas albumin showed the smallest change (1.3 mV). The distribution of zeta potentials for each isolated protein at 25 and at 37 °C are shown in the ESM, Fig. S2.

The particle-by-particle nature of TRPS allows for individual particles to be analysed, as well providing a measure of the spread in values across the sample population. Figure 3 depicts the zeta potential versus particle size plots for the given sample populations summarised in Fig. 2. Note here that each data point in Fig. 3 is representative of a single particle.

Whilst the distribution of the values does not change as the incubation temperature increases, the shift in mean zeta potential as the incubation temperature was significant. This shift may be due to the affinities of the proteins for the particle surface being affected by the incubation temperature. Previous studies have found that negative particles have maximum protein adsorption at 15, 35 and 37 °C [67] and explain why the CPC200s incubated at 37 °C in each protein medium shifted to a smaller zeta potential value more so than those incubated at 25 °C. When proteins have a higher affinity to the particle surface, there is either the formation of a robust hard corona, or slower release of the proteins from the surface once absorbed. The hard corona layer will alter the particle surface chemistry and will result in a slower particle translocation velocity through the pore due to shielding of the negative particle surface, which consequently results in a smaller zeta potential value. Interestingly, at the 25 °C incubation (Fig. 3a), the γ-globulin and particularly the fibrinogen sample showed a wider spread of data than those samples incubated at 37 °C (Fig. 3b). Figure 3b also shows that at elevated temperatures, a thicker protein corona layer is formed resulting in an increase in particle size. These results suggest the protein binding kinetics may differ as a function of temperature. The population spread may be wider at lower temperatures as the proteins may not have reached maximum levels of adsorption to the particle surface at 25 °C [67], also supporting the small changes in mean zeta potential at 25 °C demonstrated in Fig. 2.

**Fig. 4:**
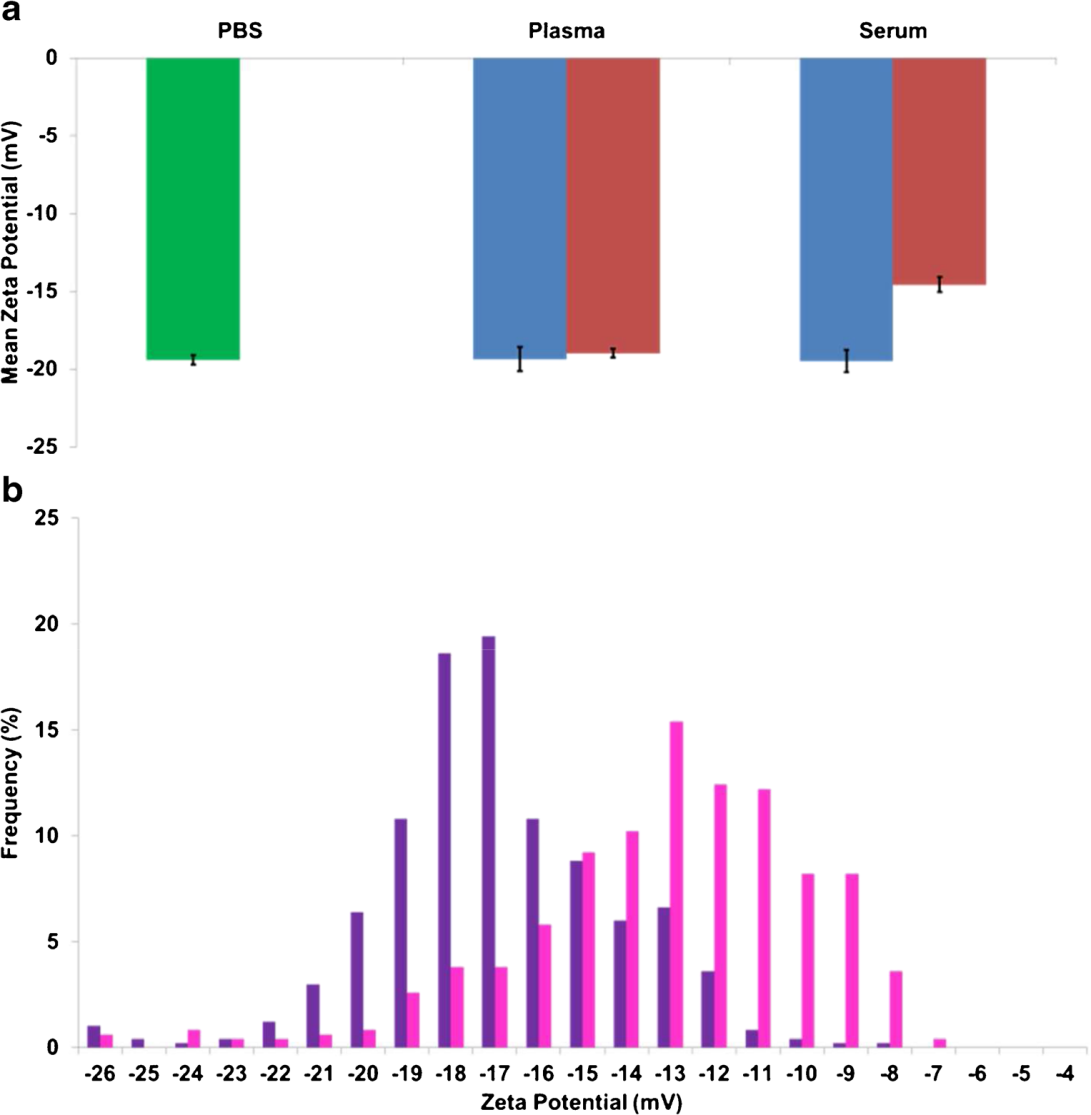
(**a**) Mean zeta potential (mV) versus incubation medium. Comparison of CPC200 particles incubated in PBS (*green*), plasma and serum for 10 min at 25 °C (*blue*) and 37 °C (*red*). Error bars are representative of the st.dev where *n* = 3. (**b**) Frequency (%) versus zeta potential (mV). Zeta potential distributions for CPC200s incubated for 10 min at 37 °C in plasma (*purple*) and serum (*pink*). Repeat datasets for CPC200s incubated in both plasma and serum at 37 °C for 10 min are illustrated in ESM Fig. S3 and are compared to a zeta potential distribution of CPC200s in PBS only

Monitoring individual protein–nanoparticle interactions is interesting but becomes more complex in a medium containing a protein mixture, such as plasma or serum. Both plasma and serum are extracted from blood samples but contain a different composition of proteins. Relevant to this study, serum contains albumin, γ-globulin and apolipoproteins. Plasma has a similar protein composition to serum, but also contains clotting factors such as fibrinogen. Figure 4 shows the measured zeta potentials of CPC200s in PBS and of CPC200s incubated in plasma and serum for 10 min at both 25 and 37 °C.

As seen in the isolated fibrinogen and γ-globulin samples above, only small changes in zeta values were observed for both plasma and serum at 25 °C. Interestingly, at the elevated incubation temperature of 37 °C, the plasma still did not appear to show a significant difference in zeta potential, whereas the sample in serum showed a reduction in zeta potential of 5.9 mV. The most prominent difference between plasma and serum is the presence of clotting factors in plasma; this will have an inherent effect on the protein corona structure and resulting interactions with the particle surface [63]. Protein corona formation is complex in physiological environments as it consists of the simultaneous binding of numerous proteins to the particle surface creating both protein-nanoparticle interactions as well as protein–protein interactions [63].

**Fig. 5:**
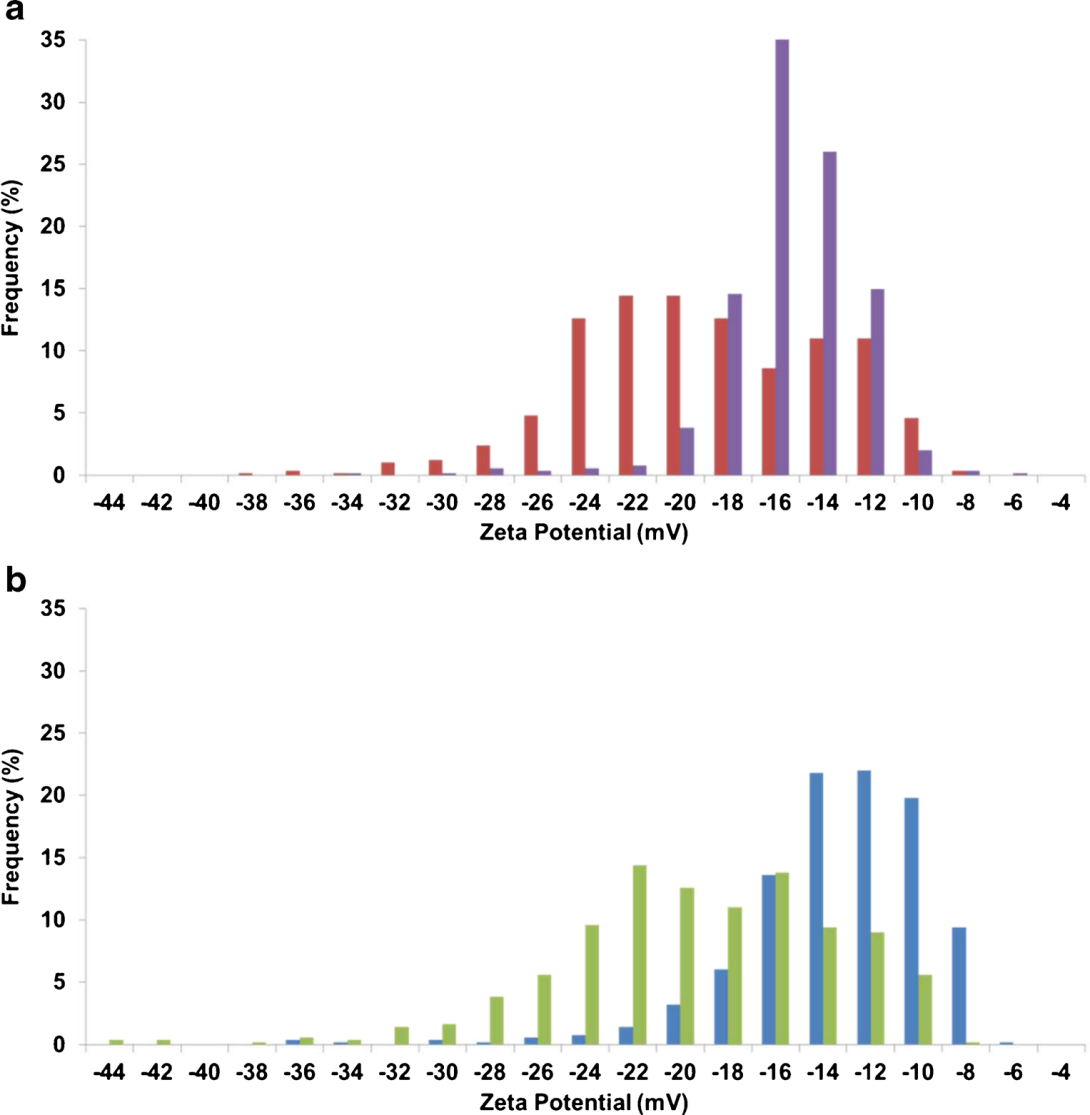
(**a**) Zeta potential distributions for CPC200s incubated in plasma for 10 min at 25 °C (*red*) and 37 °C (*purple*). (**b**) Zeta potential distributions for CPC200s incubated in serum for 10 min at 25 °C (*green*) and 37 °C (*blue*)

Proteins within plasma and serum are undergoing a competitive binding assay to the particle’s surface, and proteins of higher concentration and/or affinity will bind to the particle surface more rapidly at the first instance. Protein–protein interactions are also common in plasma and serum samples, and some proteins will have a higher affinity to a subsequent protein over the particle surface. Zeta potential distribution as a function of temperature for the particles incubated with plasma and serum samples are shown in Fig. 5.

When the incubation temperature was increased, the zeta potential for both particles in plasma and serum were smaller. The advantage of distribution studies of a sample population is the discrete differences that can be identified, that cannot be determined immediately from mean values. For example, in Fig. 5a, the distribution shape of the particles incubated with plasma at 25 °C (red) is almost twice as wide as the distribution for 37 °C (purple), yet the mean values only changed by 0.4 mV between temperatures, a negligible difference. The difference in distribution shape can be reflected using median skewness values. The median skewness values for a given sample population of particles incubated in plasma were 0.111 and −0.065 for incubation temperatures of 25 and 37 °C, respectively. Particles in serum showed the same effect and as the incubation temperature was increased, the median skewness values decreased from −0.105 (25 °C) to −0.343 (37 °C).

**Fig. 6:**
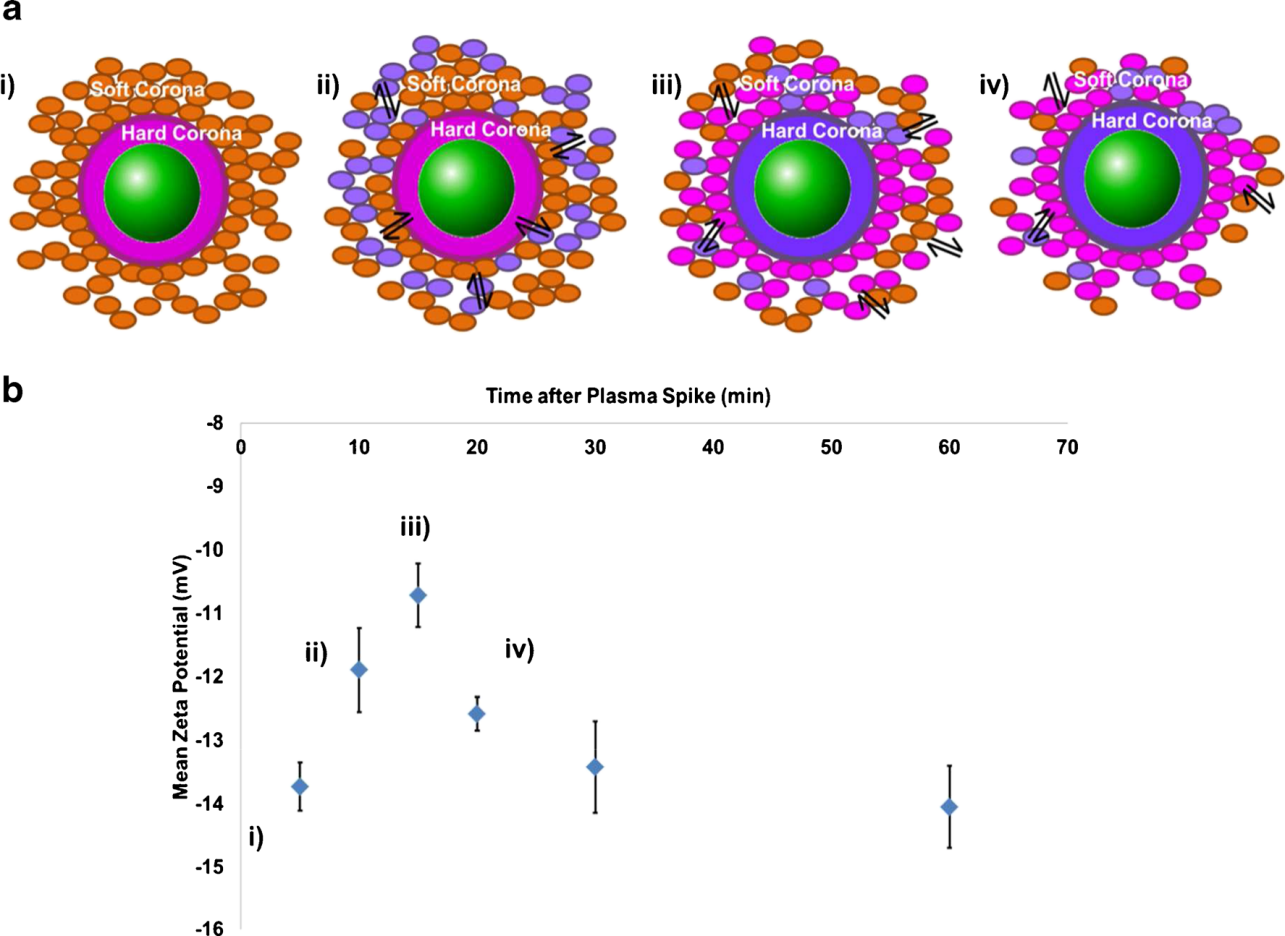
The effect of spiking a sample of CPC200s incubated in serum with plasma. (**a**) Visual representation of the effect of protein displacement and exchange within a protein corona system. (**i**) Protein corona formed by particle incubation in serum, (**ii**) introduction of plasma proteins to sample, (**iii**) displacement of hard corona proteins due to proteins of higher affinities and exchange of soft corona proteins, (**iv**) Depletion of soft corona layer as proteins dissociate from loose protein-protein interaction. (**b**) Particles were incubated in serum for 10 min and then spiked with 5 % (*v*/*v*) plasma. Zeta potentials were measured at 5, 10, 15, 20, 30 and 60 min. (**i**)–(**iv**) indicate the shift in zeta potential as a result of the effects described in (**a**)

The protein–nanoparticle interactions in plasma and serum were evidently varied, and to investigate this further, we completed a plasma spiking experiment. This aimed to ascertain if the soft corona formed in the plasma would reorganise in the presence of serum proteins. Figure 6 shows the effect on zeta potential as plasma (5 % *V*/*V*) was used to spike samples containing nanoparticles in serum at various time intervals.

Plasma protein adsorption onto a particle surface is due to the Vroman effect and is defined as the constant change in protein composition based on continuous adsorption and desorption at an interface [68]. There can be both faster and slower stages in this effect dependent on the protein. For example, albumin, γ-globulin and fibrinogen are all proteins that will adsorb rapidly onto a surface based on their high abundances, but are generally replaced by apolipoproteins in a matter of seconds [69] due to their fast dissociation properties. Apolipoproteins, however, although of low abundance, have a much slower dissociation constant and will therefore remain on the potential surface for longer [70]. As with a lot of nanoparticle-based assays, there may be an element of competition between proteins in binding to the nanoparticle surface that will affect the protein corona structure as displacement and exchange reactions may then take place over time. As the hard corona involves the higher affinitive proteins, this should remain adsorbed onto the nanoparticle surface over time and during any biophysical event that may occur [63]. The soft corona involves much weaker protein interactions in the system and will therefore dissociate more rapidly and protein exchange will occur much more readily. This effect is dependent on the relative protein concentrations of all proteins present in the plasma and serum samples. It is well known that protein concentration has a significant effect on the formation of a protein corona when incubated with nanoparticles [55, 71]; when a protein is of high concentration in a given sample, that protein will initially occupy the nanoparticle surface and form a protein corona [55] at a potentially faster rate than those of lower concentrations that may be later exchanged for those at a lower concentration but higher affinity. This effect also depends on the nanomaterial and there have been cases where proteins that have adsorbed first have had the longest residence time [72].

The first measurement was taken after the plasma had been introduced to the serum sample for 5 min. Between 5 and 10 min of the plasma being present (Fig. 6(i-ii)), the zeta potential of the particles was reduced. This is due to the addition of proteins into the sample, a higher concentration of proteins interacting with the particles will result in a slower pore translocation velocity, hence the reduced zeta potential. Figure 6(iii) shows that after 15 min, the zeta potential was reduced to its lowest measured value in this experiment. This is due to some of the plasma proteins displacing those from serum that may have reversibly bound to the particle surface as part of a hard corona layer. The plasma proteins may have been of a higher affinity to those present in the serum sample and therefore form the new hard corona layer [56, 62]. After 20 min and gradually onto 60 min (Fig. 6(iv)), the particle zeta potentials became more negative, indicating an increase in particle translocation velocity through the pore. We attribute this result to the weak interactions of the soft corona layers. For example, once the plasma proteins have potentially displaced those in the original hard corona, the displaced proteins will form part of the soft corona and be part of weaker protein–protein interactions. Over time, the soft corona proteins will dissociate more readily away from the particle due to their loose interactions [73], reducing the protein coverage around the particle and thus resulting in a larger zeta potential. The zeta potential becomes larger after this process as there are less bound proteins surrounding the particle to reduce the particle’s translocation velocity. The faster the particle can traverse the pore, the larger the zeta potential value. This is of particular interest as it gathers valuable information on how the different compositions of plasma and serum proteins in a blood sample would affect a nanoparticle and how they behave differently when isolated and in a mixture.

## Conclusions

We have demonstrated the effects of more prominent proteins found in protein coronas individually (isolated in PBS) and within their natural environment (within plasma and serum samples) on carboxylated polystyrene nanoparticle surfaces. Protein–nanoparticle interactions involved in the formation of a protein corona have been found to be protein dependent at 25 °C, as well as temperature dependent for each studied protein. Significant changes in particle zeta potentials were observed when all of the proteins interacted with the nanoparticles at 37 °C. TRPS technology has enabled the provision of single particle analysis, as well as information on the zeta potential distributions amongst a given sample population in all experiments carried out, a more detailed insight than some other previously used ensemble techniques. We have found that although a stable hard and soft corona can be formed around particles in serum, we can also track various protein displacement and exchange processes occurring when plasma proteins are introduced to these samples. This has provided more detailed information on the affinities and reaction kinetics of protein coronas dependent on their biological medium and incubation conditions. A further understanding of protein–nanoparticle interactions in complex matrices and in physiological conditions is proving useful for advances in biotechnological assays and therapeutics.

## Electronic supplementary material

Below is the link to the electronic supplementary material.

ESM 1 (PDF 862 kb)
