# Supplementary material for: Characterisation of the protein corona using tunable resistive pulse sensing: determining the change and distribution of a particle’s surface charge
Source: Anal Bioanal Chem. 2016 Jun 10;408(21):5757–68. doi: 10.1007/s00216-016-9678-6 (PMC4958399; doi:10.1007/s00216-016-9678-6)
Supplement: Supplementary file 1 — (PDF 862 kb) [file 216_2016_9678_MOESM1_ESM.pdf]

Analytical and Bioanalytical Chemistry

Electronic Supplementary Material

Characterisation of the protein corona using tunable resistive pulse sensing: determining the change and distribution of a particle's surface charge

Emma L. C. J Blundell, Matthew J. Healey, Elizabeth Holton, Muttuswamy Sivakumaran, Sarabjit Manstana, Mark Platt

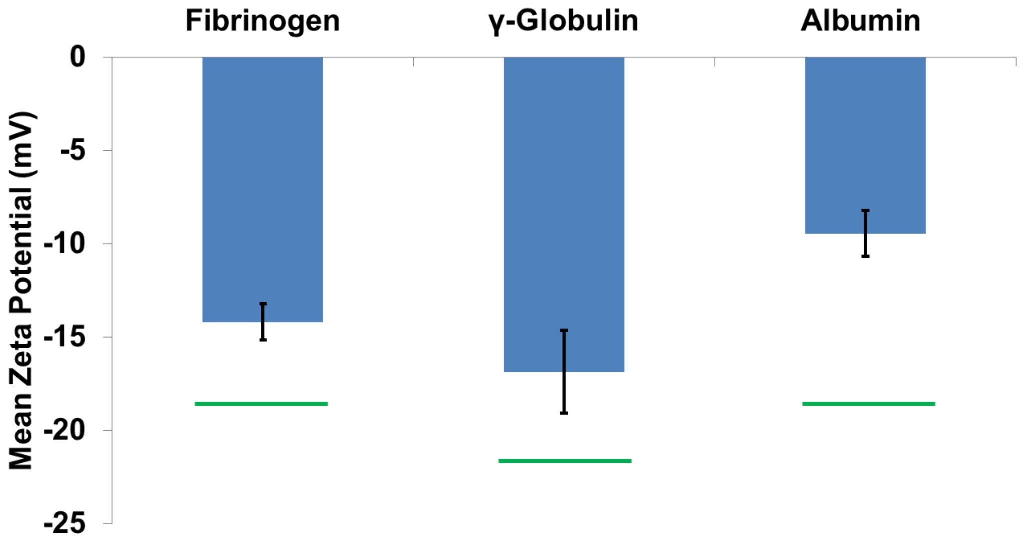

Fig. S1 Mean zeta potential (mV) vs protein particles incubated with. The blue dataset show results for a 10 minute particle incubation at 25°C and for all the proteins at a concentration of 5 g/L. The green lines represent the measured mean zeta potential for particles in PBS that were run after each protein sample to show the relative change in zeta potential once the proteins had been incubated with the particles. Error bars are representative of the st.dev where n=3

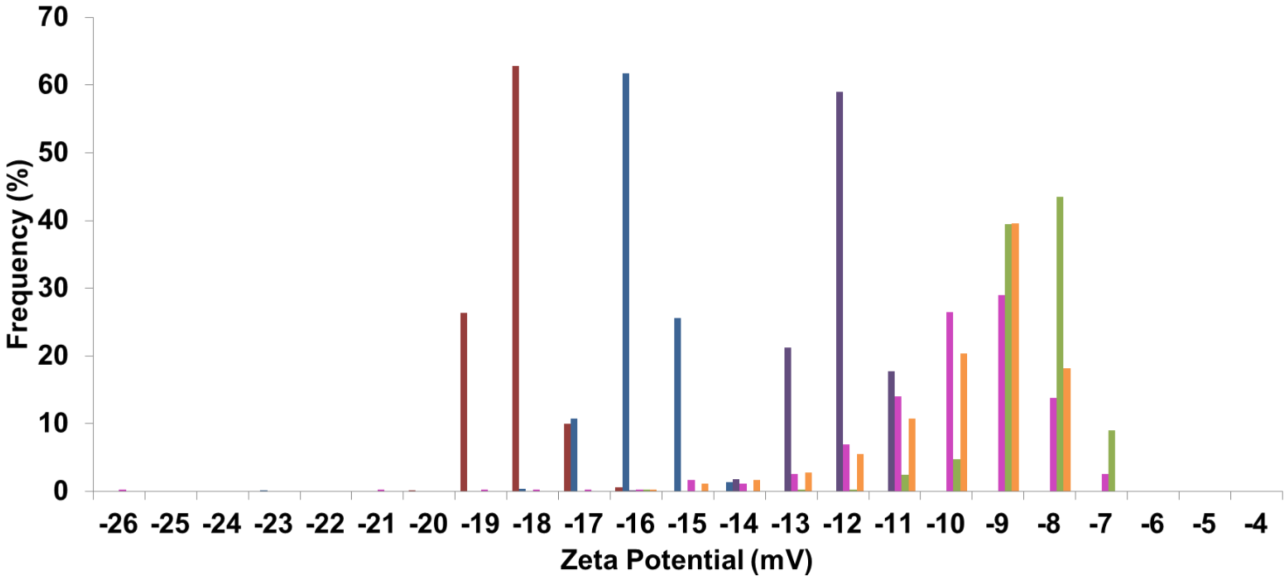

Fig. S2 Zeta potential distributions for particles incubated at 25°C with fibrinogen (red), γ-globulin (blue), albumin (purple); and particles incubated at 37°C with fibrinogen (pink), γ-globulin (green), and albumin (orange)

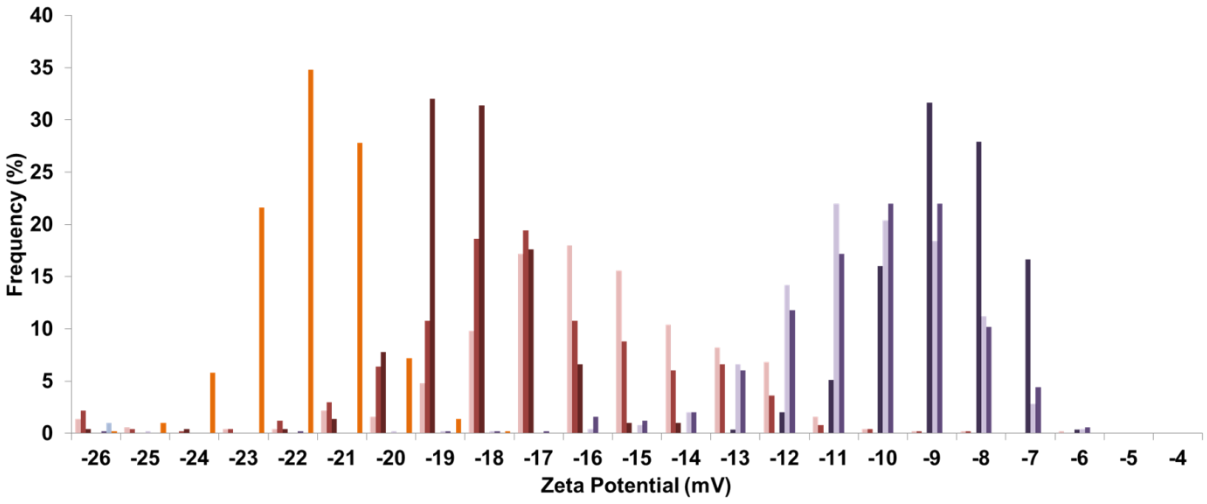

Fig. S3 Repeat datasets for zeta potential distributions for particles incubated at 37°C with PBS (orange), plasma (red datasets), and serum (purple datasets)
